# Functional characterization of FBXL7 as a novel player in human cancers

**DOI:** 10.1038/s41420-022-01143-w

**Published:** 2022-07-29

**Authors:** Yue Wang, Xiao Shen, Longyuan Gong, Yongchao Zhao, Xiufang Xiong

**Affiliations:** 1 Cancer Institute of the Second Affiliated Hospital, Zhejiang University School of Medicine, Hangzhou, Zhejiang People’s Republic of China; 2 Institute of Translational Medicine, Zhejiang University School of Medicine, Hangzhou, Zhejiang People’s Republic of China; 3 Department of Hepatobiliary and Pancreatic Surgery, the First Affiliated Hospital, Zhejiang University School of Medicine, Hangzhou, Zhejiang People’s Republic of China; 4 Zhejiang Provincial Key Laboratory of Pancreatic Disease, the First Affiliated Hospital, Zhejiang University School of Medicine, Hangzhou, Zhejiang People’s Republic of China; 5 Cancer Center, Zhejiang University, Hangzhou, Zhejiang People’s Republic of China

**Keywords:** Ubiquitin ligases, Ubiquitylation

## Abstract

F-box and leucine-rich repeat protein 7 (FBXL7), an F-box protein responsible for substrate recognition by the SKP1-Cullin-1-F-box (SCF) ubiquitin ligases, plays an emerging role in the regulation of tumorigenesis and tumor progression. FBXL7 promotes polyubiquitylation and degradation of diverse substrates and is involved in many biological processes, including apoptosis, cell proliferation, cell migration and invasion, tumor metastasis, DNA damage, glucose metabolism, planar cell polarity, and drug resistance. In this review, we summarize the downstream substrates and upstream regulators of FBXL7. We then discuss its role in tumorigenesis and tumor progression as either an oncoprotein or a tumor suppressor, and further describe its aberrant expression and association with patient survival in human cancers. Finally, we provide future perspectives on validating FBXL7 as a cancer biomarker for diagnosis and prognosis and/or as a potential therapeutic target for anticancer treatment.

## Facts

- FBXL7 is involved in the regulation of many biological processes by targeting diverse substrates for polyubiquitylation and subsequent degradation via proteasome.
- FBXL7 plays an emerging role in the regulation of tumorigenesis and tumor progression.
- FBXL7 expression is controlled by several factors at the transcriptional, post-transcriptional, and post-translational levels.
- Alterations in FBXL7 expression found in human cancers are associated with tumor progression, patient survival, and drug resistance.

## Open questions

- How FBXL7 promotes tumorigenesis and tumor progression in certain cancer types, given that the substrates identified thus far are mainly oncoproteins?
- What are the upstream signalings/kinases responsible for substrate phosphorylation to promote FBXL7-mediated substrate degradation, thereby regulating biological processes, including tumorigenesis?
- What is the role of FBXL7, either as an oncoprotein or as a tumor suppressor, in tumorigenesis under in vivo physiological settings?
- Does FBXL7 serve as a cancer biomarker for diagnosis and prognosis and/or as a potential therapeutic target for anticancer treatment?

## Introduction

Proteolysis via the ubiquitin-proteasome system (UPS) is required to maintain protein homeostasis in eukaryotic cells. By targeting proteins for polyubiquitylation and subsequent degradation by the 26S proteasome, the UPS precisely regulates many physiological and pathological processes, including cell cycle regulation, apoptosis, genomic stability, signal transduction, development, immune response, and tumorigenesis [1–4]. Protein ubiquitylation, a process that covalently attaches a polyubiquitin chain to a substrate, is a three-step enzymatic cascade reaction sequentially catalyzed by E1 ubiquitin-activating enzymes, E2 ubiquitin-conjugating enzymes, and E3 ubiquitin ligases. Of the more than 600 ubiquitin ligases, cullin-RING ligases (CRLs) are one of the largest families and mediate approximately 20% of cellular protein degradation via the UPS [5–7]. CRL1, a well-studied member of CRLs and also known as SKP1-Cullin-1-F-box protein (SCF), comprises cullin-1, a scaffold protein, RBX1, a RING protein, S-phase-kinase-associated protein 1 (SKP1), an adaptor protein, and F-box protein, a substrate recognition receptor [8]. F-box proteins, characterized by a conserved F-box motif of 42–48 amino acid, bind to the adaptor protein SKP1 for the assembly of SCF ligase complexes [9, 10]. There are ~70 F-box proteins encoded by the human genome, which are further classified into three subfamilies by the variable region at the carboxyl-terminus: the FBXW subfamily (containing 3–8 WD-40 domains), FBXL subfamily (containing 3–12 leucine-rich repeats (LRR)), and FBXO subfamily (containing other domains, such as proline-rich domains or kelch repeats) [11–13]. F-box proteins recognize and bind to various substrates via the WD/LRR motif, determining the substrate specificity of SCF ubiquitin ligases.

**Fig. 1:**
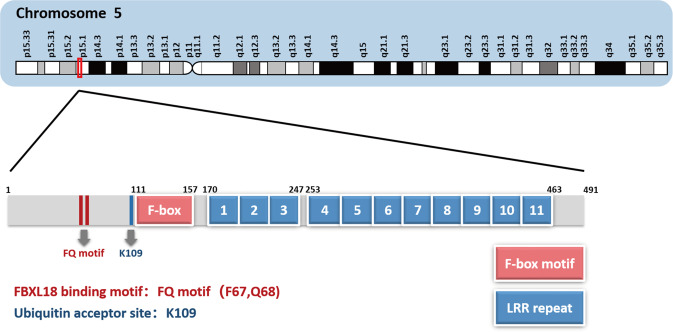
The domain structures of FBXL7. *FBXL7*, located on chromosome 5p15.1, encodes a 491 amino-acid protein that consists of an F-box motif for SKP1 binding and 11 LRR repeats at the C-terminus. The FQ motif for FBXL18 binding and ubiquitination modification site on K109 are indicated. LRR: leucine-rich repeats.

Although the FBXL subfamily, the largest subfamily of F-box proteins, consists of 22 members (FBXL1–FBXL22), very few members are well characterized like SKP2 (FBXL1) [11, 14]. F-box and leucine-rich repeat protein 7 (FBXL7), also known as FBL6, FBL7, and KIAA0840, contains 491 amino acids and is encoded by the human *FBXL7* gene located on chromosome 5p15.1 (Fig. 1). It is composed of a region rich in serine at the N-terminus, an F-box motif, and 11 LRRs at the C-terminus (Fig. 1) [15]. FBXL7 expression at mRNA levels has low specificity in human normal tissues (https://www.proteinatlas.org). Additionally, FBXL7 protein is highly evolutionarily conserved, with 98% identity between the sequences of human and mouse FBXL7 proteins, implying its biological importance [16–18]. Indeed, homozygous deletion of exon 3 in the human *FBXL7* gene encoding the F-box motif and three of the 11 LRRs, is involved in Hennekam syndrome, an autosomal recessive disorder characterized by lymphangiectasia, lymphedema, facial dysmorphism, and intellectual disability [19]. In addition, accumulated studies have been shown that FBXL7 regulates various biological processes, including apoptosis [16, 18, 20], proliferation [16, 21, 22], mitochondrial function [20], epithelial-mesenchymal transition (EMT) [23, 24], glucose metabolism [25], DNA damage [26], endothelial function [27], allergic inflammatory response [28, 29], embryonic development [30], planar cell polarity [31, 32], and drug resistance [33–35]. Therefore, FBXL7 is involved in various human diseases, including asthma [28, 29, 36, 37], atopy [38], Alzheimer’s disease [39], acute urticaria/angioedema [40], rheumatoid arthritis [41], Parkinson’s disease [42], chronic obstructive pulmonary disease [26], form-deprivation myopia [43], and cancer [23, 24, 35]. In this review, we summarize the downstream substrates and upstream regulators of FBXL7 and describe its aberrant expression in human cancers, with emphasis on its emerging roles in the regulation of tumorigenesis and tumor progression.

## Substrates of FBXL7

**Fig. 2:**
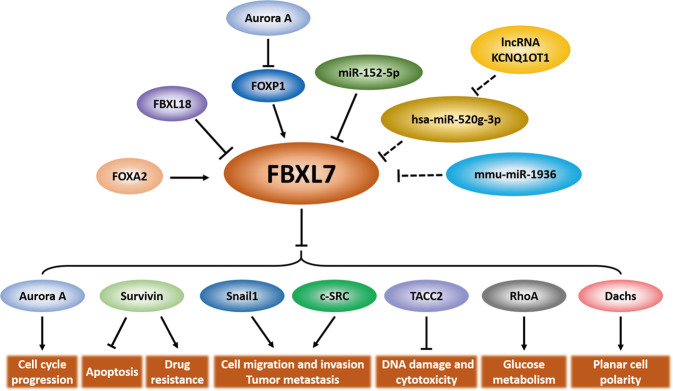
The upstream regulatory factors and the downstream substrates of FBXL7. FBXL7 is regulated by several upstream factors and targets various substrates for ubiquitination and degradation to regulate multiple biological processes. Dashed lines: The lncRNA KCNQ1OT1-hsa-miR-520g-3p axis and mmu-miR-1936 are predicted to regulate FBXL7 by bioinformatic analysis, which requires further experimental confirmation.

As a component of the SCF^FBXL7^ ligase, FBXL7 plays a key role in the recognition, recruitment, and binding of diverse substrates for targeted ubiquitylation and degradation, thus regulating cell cycle progression [16, 21, 22], apoptosis [16, 18, 20], cell migration, invasion, metastasis [23, 24], glucose metabolism [25], DNA damage [26], and drug resistance [33–35] (Fig. 2).

### Aurora A

Aurora A, a key player in the formation of mitotic spindle and chromosome segregation, was the first substrate of FBXL7 identified in 2012 [16]. Of 18 F-box proteins, including nine FBXL and nine FBXW subfamily members, FBXL7 was the only one that reduced the protein level of Aurora A upon overexpression in transformed murine lung epithelial cells [16]. Overexpression of FBXL7 promotes the polyubiquitylation and turnover of Aurora A in a time- and dose-dependent manner, but has no effects on its family member Aurora B, which shares 71% sequence identity with Aurora A, indicating the specificity of substrate recognition by FBXL7 [16]. FBXL7 colocalizes with Aurora A and targets it for degradation on the centrosome during mitosis [16]. Biologically, FBXL7 ectopic expression disrupts mitotic spindle formation and induces polyploidy, leading to G2/M arrest and apoptosis [16]. Interestingly, Aurora A inhibits FBXL7 transcription in gastric cancer cells [33]. Thus, the crosstalk between Aurora A and FBXL7 may precisely control cell cycle progression.

### Survivin

Survivin, one of the inhibitor of apoptosis (IAP) family members, plays a critical role in apoptosis inhibition and mitosis progression. The expression level of Survivin is elevated in a variety of cancers and is associated with worse prognosis and chemotherapeutic resistance [44, 45]. FBXL7 was identified as a ubiquitin ligase that mediates polyubiquitylation of Survivin to promote its degradation, causing mitochondrial dysfunction and apoptosis [20]. Survivin binds to FBXL7 with the help of its Glu126 residue. Mutation of Glu126 to alanine not only abolished the binding ability of Survivin to FBXL7 but also markedly increased its stability [20]. Overexpression of wild-type Survivin or the FBXL7 binding mutant (E126A) remarkably rescued mitochondrial dysfunction caused by FBXL7 [20]. In addition, Thr34 appears to be critical for FBXL7-mediated Survivin turnover as well [34]. Conversely, mutation of Thr34 to alanine promotes Survivin polyubiquitylation and shortens its protein half-life upon treatment with xanthohumol, a natural compound that substantially inhibits the survival of oral squamous cell carcinoma (OSCC) cells [34]. Moreover, xanthohumol suppresses Thr34 phosphorylation by inhibiting AKT activation to promote FBXL7-mediated Survivin destabilization, leading to apoptosis [34]. It is well-known that phosphorylation of substrates is crucial for them to be recognized by and bind to F-box protein [13], but how Thr34 dephosphorylation induces Survivin degradation mediated by FBXL7 remains unknown. Consistently, FBXL7 contributes to xanthohumol-mediated inhibition of OSCC xenograft tumor growth by targeting Survivin degradation [34]. Furthermore, FBXL7-mediated ubiquitylation and degradation of Survivin are regulated by Aurora A [33]. VX-680, a small molecular inhibitor of Aurora A, reduces Survivin levels in a proteasome-dependent manner, indicating that FBXL7-mediated Survivin degradation relies on the kinase activity of Aurora A [33]. Mechanistically, as mentioned above, Aurora A activation represses FBXL7 transcriptional expression causing Survivin accumulation, thus contributing to gastric cancer cell growth and resistance to doxorubicin [33].

### c-SRC

The Src family kinase c-SRC, a non-receptor-type tyrosine kinase, plays a critical role in tumor progression and metastasis [46, 47]. Recently, c-SRC was identified as a substrate for SCF^FBXL7^ ubiquitin ligase [24]. FBXL7 recognizes c-SRC upon its phosphorylation at Ser104 within its SH3 domain (a.a.102–116), and then targets the active c-SRC for polyubiquitylation and degradation [24]. Silencing of FBXL7 upregulates c-SRC levels, activates downstream signaling cascades, and subsequently induces the expression of mesenchymal markers to promote EMT, leading to the induction of cell motility, migration, and invasion, which can be reversed by c-SRC inhibition or depletion [24, 48]. More importantly, FBXL7 depletion strikingly increased the metastasis of pancreatic and prostate cancer cells in multiple orthotopically transplanted mouse models in a manner dependent of c-SRC [24]. Consistently, the expression level of FBXL7 is negatively correlated with the expression of c-Src in human prostate and pancreatic carcinoma tissues [24]. Furthermore, some point mutations in FBXL7 that cause the inability to degrade c-Src, were identified in various human cancers [24]. Hence, FBXL7 plays a tumor-suppressive role in preventing tumor progression, particularly metastasis, by promoting c-SRC degradation.

### Snail1

**Fig. 3:**
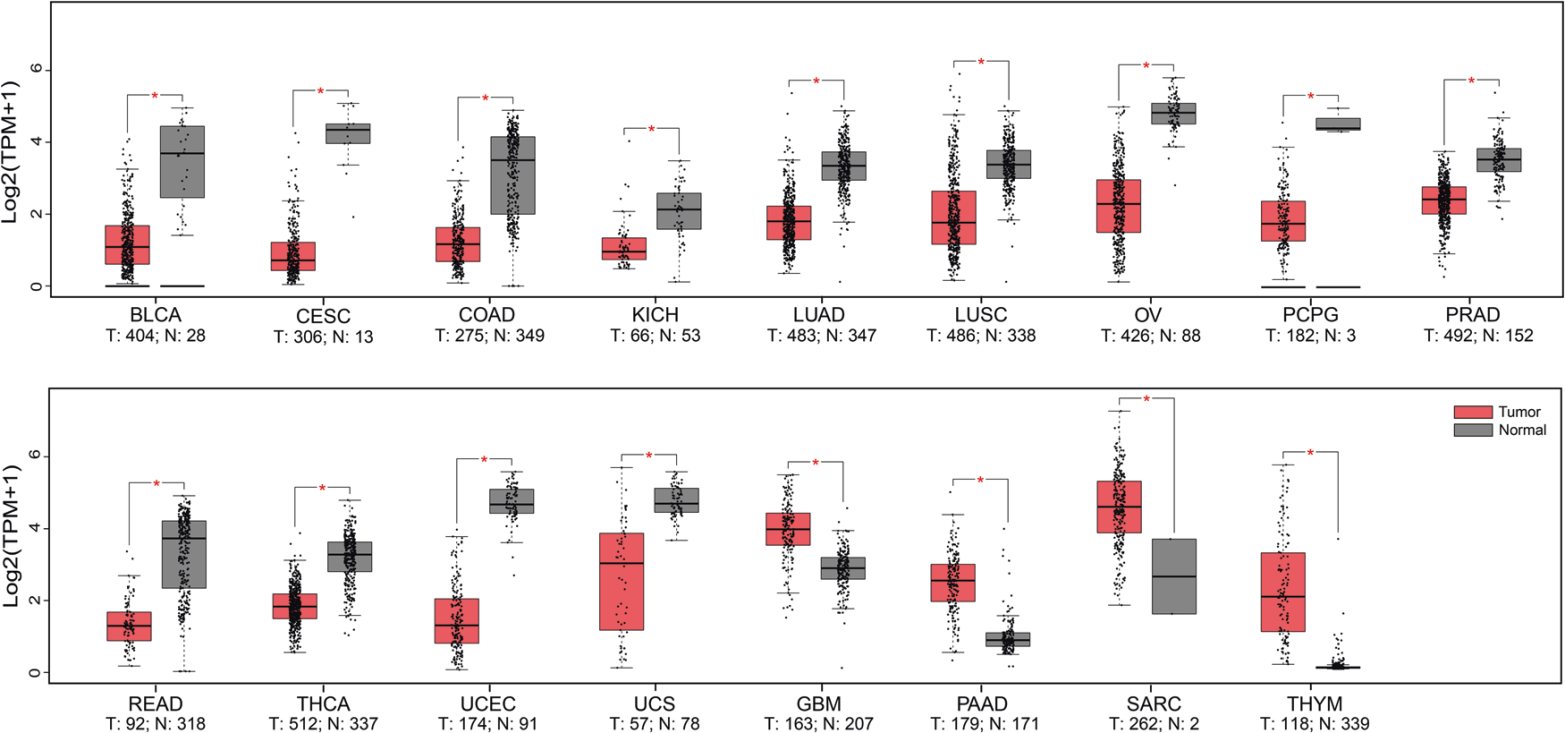
The expression levels of FBXL7 between human tumor tissues and their corresponding normal tissues. The levels of FBXL7 transcripts are markedly altered in various human cancer tissues, compared to their corresponding normal controls, based on the analysis of Gene Expression Profiling Interactive Analysis (GEPIA) database with tumor and normal tissue samples from The Cancer Genome Atlas (TCGA) and Genotype-Tissue Expression (GTEx) projects. The number of tumor (T) and normal (N) tissues is indicated. BLCA, bladder urothelial carcinoma; CESC, cervical squamous cell carcinoma and endocervical adenocarcinoma; COAD, colon adenocarcinoma; GBM, glioblastoma multiforme; KICH, kidney chromophobe; LUAD, lung adenocarcinoma; LUSC, lung squamous cell carcinoma; OV, ovarian serous cystadenocarcinoma; PAAD, pancreatic adenocarcinoma; PCPG, pheochromocytoma and paraganglioma; PARD, prostate adenocarcinoma; READ, rectum adenocarcinoma; SARC, sarcoma; THCA, thyroid carcinoma; THYM, thymoma; UCEC, uterine corpus endometrial carcinoma; UCS, uterine carcinosarcoma. TPM: transcripts per million; *p < 0.05, one-way ANOVA.

Snail1, also known as Snail, belongs to the Snail superfamily of zinc-finger transcription factors and is characterized by four consensus zinc-finger motifs responsible for Snail1 binding to the E-box motifs to regulate the transcription of downstream target genes involved in EMT, including E-cadherin, TWIST, ZEB1, collagens, fibronectin, and matrix metalloproteinases [49, 50]. As one of the critical promoting factors of EMT, the turnover of Snail1 is tightly governed by several F-box proteins in some specific cancers, including β-TrCP [51, 52] and FBXO22 [53] in breast cancer cells, FBXL14 [54] and FBXO11 [55, 56] in both breast and colon cancer cells, FBXL5 [57, 58] in breast cancer, colon cancer, gastric cancer, and pancreatic cancer cells, FBXO31 [59] in gastric cancer cells, FBXO45 [60] in prostate cancer cells, and FBXO11 [56] and FBXW7 [61, 62] in lung cancer cells. In human pancreatic cancer BxPC-3 and PANC-1 cells, FBXL7 interacts with Snail1 and promotes its ubiquitylation and degradation via proteasome to suppress EMT, thereby repressing cell migration and invasion. Silencing FBXL7 increases Snail1 levels and tumor metastasis in vivo [23]. Surprisingly, FBXL7 expression in pancreas appears to be lower than that in other specific tissues, where Snail1 is targeted by other E3 ligases, including colon, breast, prostate, and lung tissues (https://www.proteinatlas.org). However, FBXL7 expression both at mRNA and protein levels in pancreatic cancer specimens was further lower than that in matched tumor-adjacent tissues [23]. Interestingly, compared with their corresponding normal tissues, FBXL7 expression is significantly lower in colon adenocarcinoma, lung adenocarcinoma, lung squamous cell carcinoma, and prostate adenocarcinoma (Fig. 3), implying its potential regulation of Snail1 turnover in these specific cancer tissues. However, silencing FBXL7 had no evident effect on the protein levels of Snail1 in either SW620 colon cancer cells or MCF-7 breast cancer cells [57], indicating that FBXL7-mediated Snail1 turnover likely occurs in a manner dependent of specific cell context.

### TACC2

Transforming acidic coiled-coil-containing protein 2 (TACC2), one of the TACC family members consisting of a conserved TACC domain at the C-terminus, plays a key role in the regulation of centrosome and microtubule dynamics during mitosis [63]. High TACC2 expression found in human cancers, including breast cancer [64], hepatocellular carcinoma [65], and prostate cancer [66], is correlated with poor prognosis, suggesting its tumor-promoting potential. Recently, compared with the smokers without chronic obstructive pulmonary disease (COPD), TACC2 protein levels were found to be reduced in the lung tissues of smokers with COPD [26]. TACC2 plays a key role in protecting lung epithelial cells from DNA damage and cell death induced by cigarette smoke extract. Mechanistically, cigarette smoke exposure increased the phosphorylation of TACC2 at Ser304/399 and triggered the interaction of TACC2 with FBXL7, which in turn promoted K48-linkage polyubiquitylation and degradation of TACC2, leading to the induction of DNA damage and apoptosis, which probably contributes to lung inflammation and emphysema [26]. Given the importance of dysregulation of DNA damage repair and cell cycle checkpoints as cancer hallmarks [67], it is plausible that FBXL7 might regulate tumorigenesis by targeting TACC2 for degradation.

### RhoA

RhoA, one of the Rho small-GTPase family members, is well-known for its regulation of cytoskeleton dynamics to modulate cell morphology, migration, and invasion, and its expression level is markedly associated with metastasis and poor prognosis in human cancers, including breast [68], colorectal [69], and gastric cancers [70]. RhoA also takes part in insulin signaling and the maintenance of glucose homeostasis through its downstream effectors, such as ROCK that mediate glucose transport stimulated by insulin [71]. Interestingly, Fbxl7 has been validated to regulate the maintenance of glucose metabolism in mouse liver [25]. Specifically, recombinant adeno-associated virus-mediated depletion of hepatic Fbxl7 reduces glucose tolerance, whereas its overexpression improves glucose tolerance in mice [25]. Mechanistically, Fbxl7 induces the polyubiquitylation and degradation of RhoA and promotes insulin signaling in HepG2 hepatocellular carcinoma cells [25]. Therefore, FBXL7 may regulate glucose metabolism by destroying RhoA.

### Dachs

FBXL7 has also been demonstrated to modulate planar cell polarity (PCP) by regulating the Dachsous-Fat-Dachs system in *Drosophila* [31, 32]. Dachs, an atypical myosin, is a critical effector of both Dachsous and Fat, two atypical cadherins that bind to each other to form cell-cell junctions [31]. Fbxl7 is translocated to the plasma membrane at the proximal side via the interaction with the intracellular D domain of Fat and promotes the polyubiquitylation and proteasomal degradation of Dachs, restricting the localization of Dachs to the distal side, where it forms a protein complex with Dachsous [31, 32]. Consistently, Fbxl7 is distributed at the proximal edge of cells, where it is co-localized with Fat via the LRR repeats of Fbxl7 [31]. As a result, mutations in either *Fbxl7* or *Fat* in *Drosophila* show similar defects in proximodistal patterning [31]. In addition, Fbxl7 can promote ubiquitylation of the intracellular domain of Dachsous in cell culture settings. However, it still needs to be investigated whether and how Fbxl7 regulates the levels and planal polarized localization of Dachsous in vivo [32]. Consistently, FBXL7 might be involved in cell polarity regulation by binding to human PARD3, PARD3B, and PARD6G, especially given that PARP6G levels are induced by the treatment of MLN4924, a potent inhibitor of SCF ubiquitin ligases [72]. Notably, a homozygous mutation in the human *FBXL7* gene to delete three LRRs and the F-box motif was found in a patient with Hennekam syndrome, who displayed no abnormalities in known disease genes, including *FAT4*, a human ortholog of *Drosophila Fat*, suggesting that FBXL7 may also function in a pathway involving FAT4 in humans [19]. In addition, overgrowth of wings observed in *Drosophila* without functional Fbxl7 indicates that Fbxl7 negatively regulates tissue growth [31].

## Regulation of FBXL7

Several studies have shown that FBXL7 expression is controlled by various factors at the transcriptional, post-transcriptional, and post-translational levels (Fig. 2).

### Transcriptional regulation

Promoter methylation plays a crucial role in the regulation of FBXL7 expression. Among the 69 genes encoding human F-box proteins, *FBXL7* promoter is the most hypermethylated in tumor tissues, based on the analysis of pan-cancer promoter methylation using MethHC and TCGA databases [24]. Hypermethylation of *FBXL7* promoter correlates with downregulation of FBXL7 levels and advanced tumor grade in both pancreatic carcinoma and prostate cancer [24, 48]. More importantly, decitabine, an inhibitor of methylase, restores FBXL7 expression levels and inhibits cell migration and invasion, and tumor metastasis in an FBXL7-dependent manner [24]. Thus, epigenetic suppression of FBXL7 by promoter methylation is an important mechanism for downregulating the expression of FBXL7, leading to tumor progression and metastasis [24].

In addition, FBXL7 expression level is controlled by transcription factors Forkhead Box protein P1 (FOXP1) [33] and Forkhead Box protein A2 (FOXA2) [25]. Specifically, FOXP1 is recruited to multiple sites within the promoter region of *FBXL7* and transactivates the expression of FBXL7 in gastric cancer cells [33]. FOXP1-mediated FBXL7 transcriptional activation is negatively regulated by Aurora A [33]. Aurora A binds to FOXP1 at the *FBXL7* promoter and appears to phosphorylate FOXP1 at multiple putative phosphorylation sites, including S440 in its transactivation domain, resulting in the repression of FBXL7 transcription [33]. Given that FBXL7 is a subunit of ubiquitin ligase complex that targets Aurora A for degradation [16], and that Aurora A downregulates FBXL7 via FOXP1, it establishes a feed-forward loop. Thus, downregulation of FBXL7 may contribute to the overexpression of Aurora A, which is associated with worse prognosis in gastric cancer [33]. Moreover, FBXL7 repression by the Aurora A-FOXP1 axis further suppresses FBXL7-mediated Survivin ubiquitylation and subsequent degradation via proteasome, resulting in drug resistance in gastric cancer cells [33]. In addition to FOXP1, another Forkhead Box protein A2 (FOXA2) appears to regulate FBXL7 expression [25]. The protein levels of FBXL7 increased in cells overexpressing FOXA2, but decreased upon silencing of FOXA2. However, whether FOXA2 positively regulates FBXL7 at the transcriptional level requires further investigation, given that FOXA2 can interact with FBXL7 [25]. Taken together, FBXL7 expression is transcriptionally regulated by promoter methylation and transcription factors.

### Post-transcriptional regulation

Several microRNAs, including miR-152-5p [17], hsa-miR-520g-3p [42], and mmu-miR-1936 [43], are associated with FBXL7 expression. miR-152-5p represses luciferase reporter activity mediated by FBXL7 mRNA 3ʹ-UTR, thereby negatively regulating FBXL7 expression [17]. Overexpression of FBXL7 almost completely reversed the inhibition of glioma cell proliferation, migration, invasion, and enhanced glioma cell sensitivity to the anti-glioma drug temozolomide by miR-152-5p overexpression [17]. The levels of miR-152-5p were gradually downregulated, whereas FBXL7 expression was gradually upregulated with an increase in tumor grade in glioma tissues [17]. Moreover, the levels of miR-152-5p were inversely correlated with FBXL7 levels in advanced glioma tissues [17]. Therefore, the miR-152-5p-FBXL7 axis appears to play a significant role in glioma progression and in the efficacy of chemotherapeutic drugs. In addition, FBXL7 was identified as a hub gene involved in the pathogenesis of Parkinson’s disease, its expression being higher in the substantia nigra tissues of Parkinson’s disease, compared to that in normal controls [42]. FBXL7 mRNA levels are markedly associated with lncRNA KCNQ1OT1 predicted to target FBXL7 mRNA via hsa-miR-520g-3p based on the construction of a competing endogenous RNAs (ceRNA) network associated with Parkinson’s disease by database analysis [42]. Similarly, FBXL7 was also predicted to be a target of mmu-miR-1936, one of the three substantially upregulated miRNAs identified by bioinformatics analysis in mice with form-deprivation myopia [43], implying a potential role of FBXL7 in myopia development.

### Post-translational regulation

Only a few lines of evidence have documented the post-translational modifications of FBXL7, including ubiquitylation [18] and N-myristoylation [73]. FBXL7 does not undergo autoubiquitylation, whereas FBXL18 can mediate the ubiquitylation of FBXL7 at Lys109 (Fig. 1), leading to its degradation via the 26S proteasome [18]. Moreover, the N-terminal FQ motif of FBXL7 is essential for its binding to FBXL18 and its degradation, but is dispensable for its ability to form an SCF ligase complex via binding to SKP1 and to destabilize the substrate (Fig. 1) [18]. Biologically, simultaneous silencing of FBXL7 largely reverses apoptosis induction by FBXL18 knockdown [18]. Thus, FBXL7 is a substrate of the SCF^FBXL18^ ligase. In addition, by the systematic proteomics analysis of the binding proteins of the FBXL family, FBXL7 was found to interact with three deubiquitylases: USP1, USP12, and USP46 [72], implying that they may stabilize FBXL7 via deubiquitylation. Besides ubiquitylation, FBXL7 was identified to be N-myristoylated [73]. Protein N-myristoylation, a lipid modification, is a process of attaching myristate to the glycine residue of the substrate protein at its N-terminus, which plays a crucial role in the subcellular localization of substrates, signal transduction, immune response, and tumorigenesis [74, 75]. However, further studies are needed to clarify the biological impact of N-myristoylation on FBXL7.

## Functions of FBXL7 and its alteration in human cancers

FBXL7 is abnormally expressed in various cancers, including ovarian [35], pancreatic [23, 24], prostate [24], and brain cancers [17]. The single nucleotide polymorphism (SNP) rs12652447 of *FBXL7* is relevant to an increased risk of breast cancer in people carrying breast cancer gene 2 (BRCA2) mutations in breast cancer genome-wide association studies [76]. Moreover, FBXL7 was predicted to be associated with lung tumorigenesis using computational methods [77].

FBXL7 exhibits tumor-suppressive characteristics by mediating degradation of several oncogenic substrates. As mentioned above, overexpression of FBXL7 suppresses cell proliferation and causes apoptosis through destabilizing Aurora A and Survivin in lung cancer cells [16, 20]. FBXL7 knockdown promotes cell migration, invasion, and tumor metastasis via c-Src accumulation in pancreatic and advanced prostate cancers [24]. Moreover, FBXL7 can negatively regulate pancreatic cancer cell migration and invasion via targeting Snail1 for degradation [23]. Consistently, lower FBXL7 expression resulting from promoter hypermethylation predicts worse survival in patients with prostate and pancreatic cancers [24]. Additionally, both mRNA and protein levels of FBXL7 were found to be reduced in pancreatic cancer tissues, compared to those in the corresponding tumor-adjacent normal tissues [23]. To fully understand the alteration of FBXL7 expression levels in human cancers, we compared its mRNA levels in human cancer tissues with those in normal tissues using the GEPIA database (http://gepia.cancer-pku.cn/) [78]. As shown in Fig. 3, FBXL7 mRNA levels were substantially decreased in the majority of cancers, including bladder urothelial carcinoma, cervical squamous cell carcinoma and endocervical adenocarcinoma, colon adenocarcinoma, kidney chromophobe, lung adenocarcinoma, lung squamous cell carcinoma, ovarian serous cystadenocarcinoma, pheochromocytoma and paraganglioma, prostate adenocarcinoma, rectum adenocarcinoma, thyroid carcinoma, uterine corpus endometrial carcinoma, and uterine carcinosarcoma. Additionally, the human *FBXL7* gene displays rare sporadic mutations in various carcinomas of the lung, breast, liver, stomach, pancreas, large intestine, colon, rectum, and prostate [24]. Some of these mutants disabled FBXL7 to degrade substrates, including 1) P65S, P93L, and R310H, which are unable to bind to the substrate, and 2) Q271H, R353Q, T458M, R480H, R480C, and Y145*, which cannot bind to CUL1 and/or SKP1 to form a functional SCF ligase complex [24]. Thus, the downregulation or dysfunction of FBXL7 may cause the accumulation of substrates, promoting tumorigenesis and tumor progression.

**Fig. 4:**
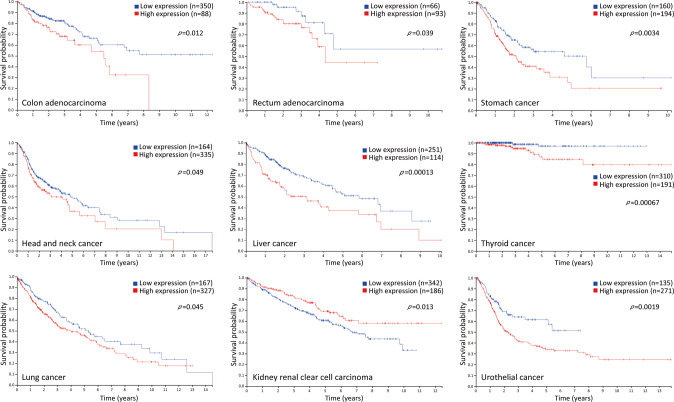
High FBXL7 expression predicts a poor patient survival in a variety of human cancers. FBXL7 expression is markedly associated with patient survival in various cancers according to the Human Protein Atlas database. Blue line: low FBXL7 expression; red line: high FBXL7 expression. The *p*-value for the log-rank test is indicated.

In contrast, in ovarian cancer patients, higher levels of FBXL7 transcripts correlate with worse overall survival and progression-free survival and also predict a higher risk of recurrence after adjuvant chemotherapy [35]. Immunohistochemical staining also confirmed that the protein levels of FBXL7 were inversely correlated with the overall survival of ovarian cancer pateints [35]. More importantly, FBXL7 levels are markedly correlated with paclitaxel resistance in ovarian cancer cells [35]. Moreover, FBXL7 level negatively correlates with the progression and survival of patients with glioma [17]. FBXL7 mRNA levels gradually increased with increasing glioma grade. FBXL7 silencing inhibits proliferation, migration, and invasion of glioma cells and sensitizes glioma cells and ovarian cancer cells resistant to chemotherapeutic drugs [17, 35]. Thus, FBXL7 may serve as a biomarker for poor prognosis and may be a therapeutic target for patients with glioma and ovarian cancer, particularly those who have undergone chemotherapy. Furthermore, we determined the association between FBXL7 expression and overall survival using the Human Protein Atlas database. As shown in Fig. 4, patients with higher levels of FBXL7 displayed a worse overall survival in various cancers, including colon adenocarcinoma, rectum adenocarcinoma, stomach cancer, head and neck cancer, liver cancer, thyroid cancer, lung cancer, and urothelial cancer, but better survival only in kidney renal clear cell carcinoma, implying the oncogenic characteristics of FBXL7.

## Conclusion and future perspectives

In conclusion, FBXL7 plays an important role in the regulation of many biological processes via targeting polyubiquitylation and degradation of diverse substrates (Fig. 2). Alterations in FBXL7 expression in human cancers and its association with tumor metastasis, patient survival, and drug resistance imply that FBXL7 may function as either an oncoprotein or a tumor suppressor. Given that the substrates identified thus far are mainly oncoproteins, the mechanism by which FBXL7 promotes tumorigenesis and tumor progression in certain cancer types is unclear. Thus, it is important to identify additional specific substrates of FBXL7, particularly those critical for the regulation of tumorigenesis, to fully understand the context-dependent roles of FBXL7 in certain cancer types. Additionally, since phosphorylation plays a crucial role in substrate recognition by FBXL7 [24, 26, 34], similar to other F-box proteins [13], exploring the upstream signaling/kinase responsible for substrate phosphorylation would help uncover the mechanisms by which FBXL7 regulates many biological processes, including tumorigenesis. Furthermore, the downregulation of FBXL7 expression in some cancers, including colon adenocarcinoma, lung cancer, ovarian cancer, rectum adenocarcinoma, and thyroid cancer, appears to be paradoxical with the survival analysis showing that higher FBXL7 levels predict a worse prognosis (Figs. 3 and 4). Thus, it is critical to clarify the causal role of FBXL7 in tumorigenesis under in vivo physiological settings. To date, no studies using *FBXL7* total knockout or conditional knockout mice have been published, although it has been known that *Drosophila* harboring *Fbxl7* loss-of-function mutations are viable and fertile and show a growth advantage [31]. Studies using genetically modified mouse tumor models that combine *FBXL7* knockout mice with oncogene activation (such as *Kras*^*G12D*^) or tumor suppressor inactivation (such as *Trp53*^*fl/fl*^, *Pten*^*fl/fl*^) are in high demand to elucidate the physiological roles of FBXL7, either as an oncoprotein or as a tumor suppressor. Moreover, since the accumulated evidence mainly supports a tumor-suppressive role of FBXL7, a better exploitation of the upstream regulation might provide potential strategies to activate FBXL7 pathway for anticancer treatment. Indeed, increasing FBXL7 levels by decitabine, an FDA-approved methylase inhibitor, was able to inhibit metastasis in orthotopically transplanted mouse models of prostate and pancreatic cancer with low FBXL7 levels due to promoter hypermethylation [24]. Another potential approach is to use synthetic lethal strategies to target specific cancers with FBXL7 loss-of-function mutations or decrease in its expression levels. For instance, it is worth exploring whether the inhibitors of Aurora A, a negative regulator of FBXL7 expression in gastric cancer cells [33], would lead to synthetic lethality in gastric cancer with decreased FBXL7 expression/function. Taken together, further investigations on FBXL7 and its mechanisms of action are required to determine FBXL7 as a cancer biomarker for diagnosis and prognosis and/or as a potential therapeutic target for anticancer treatment.

## Data Availability

Data sharing is not applicable to this article, as no datasets were generated or analyzed during the current study.

## References

[1] Nakayama KI, Nakayama K (2006). Ubiquitin ligases: cell-cycle control and cancer. Nat Rev Cancer.

[2] Rape M (2018). Ubiquitylation at the crossroads of development and disease. Nat Rev Mol Cell Biol.

[3] Senft D, Qi J, Ronai ZA (2018). Ubiquitin ligases in oncogenic transformation and cancer therapy. Nat Rev Cancer.

[4] Liu J, Qian C, Cao X (2016). Post-translational modification control of innate immunity. Immunity..

[5] Petroski MD, Deshaies RJ (2005). Function and regulation of cullin-RING ubiquitin ligases. Nat Rev Mol Cell Biol.

[6] Zhao Y, Sun Y (2013). Cullin-RING ligases as attractive anti-cancer targets. Curr Pharm Des.

[7] Cui D, Xiong X, Zhao Y (2016). Cullin-RING ligases in regulation of autophagy. Cell Div.

[8] Skaar JR, Pagan JK, Pagano M (2014). SCF ubiquitin ligase-targeted therapies. Nat Rev Drug Discov.

[9] Zhou W, Wei W, Sun Y (2013). Genetically engineered mouse models for functional studies of SKP1-CUL1-F-box-protein (SCF) E3 ubiquitin ligases. Cell Res.

[10] Wang Z, Liu P, Inuzuka H, Wei W (2014). Roles of F-box proteins in cancer. Nat Rev Cancer.

[11] Tekcham DS, Chen D, Liu Y, Ling T, Zhang Y, Chen H (2020). F-box proteins and cancer: an update from functional and regulatory mechanism to therapeutic clinical prospects. Theranostics..

[12] Nguyen KM, Busino L (2020). The biology of F-box proteins: the SCF family of E3 ubiquitin ligases. Adv Exp Med Biol.

[13] Jin J, Cardozo T, Lovering RC, Elledge SJ, Pagano M, Harper JW (2004). Systematic analysis and nomenclature of mammalian F-box proteins. Genes Dev.

[14] Mason B, Laman H (2020). The FBXL family of F-box proteins: variations on a theme. Open Biol.

[15] Ilyin GP, Rialland M, Pigeon C, Guguen-Guillouzo C (2000). cDNA cloning and expression analysis of new members of the mammalian F-box protein family. Genomics..

[16] Coon TA, Glasser JR, Mallampalli RK, Chen BB (2012). Novel E3 ligase component FBXL7 ubiquitinates and degrades Aurora A, causing mitotic arrest. Cell Cycle.

[17] Kong S, Fang Y, Wang B, Cao Y, He R, Zhao Z (2020). miR-152-5p suppresses glioma progression and tumorigenesis and potentiates temozolomide sensitivity by targeting FBXL7. J Cell Mol Med.

[18] Liu Y, Lear T, Zhao Y, Zhao J, Zou C, Chen BB (2015). F-box protein Fbxl18 mediates polyubiquitylation and proteasomal degradation of the pro-apoptotic SCF subunit Fbxl7. Cell Death Dis.

[19] Boone PM, Paterson S, Mohajeri K, Zhu W, Genetti CA, Tai DJC (2020). Biallelic mutation of FBXL7 suggests a novel form of Hennekam syndrome. Am J Med Genet A..

[20] Liu Y, Lear T, Iannone O, Shiva S, Corey C, Rajbhandari S (2015). The proapoptotic F-box Protein Fbxl7 regulates mitochondrial function by mediating the ubiquitylation and proteasomal degradation of survivin. J Biol Chem.

[21] Barral Y, Jentsch S, Mann C (1995). G1 cyclin turnover and nutrient uptake are controlled by a common pathway in yeast. Genes Dev.

[22] Kishi T, Yamao F (1998). An essential function of Grr1 for the degradation of Cln2 is to act as a binding core that links Cln2 to Skp1. J Cell Sci.

[23] Tang L, Ji M, Liang X, Chen D, Liu A, Yang G (2021). Downregulated F-Box/LRR-repeat protein 7 facilitates pancreatic cancer metastasis by regulating snail1 for proteasomal degradation. Front Genet.

[24] Moro L, Simoneschi D, Kurz E, Arbini AA, Jang S, Guaragnella N (2020). Epigenetic silencing of the ubiquitin ligase subunit FBXL7 impairs c-SRC degradation and promotes epithelial-to-mesenchymal transition and metastasis. Nat Cell Biol.

[25] Jia Y, Liu W, Bai D, Zhang Y, Li Y, Liu Y (2022). Melatonin supplementation in the culture medium rescues impaired glucose metabolism in IVF mice offspring. J Pineal Res.

[26] Mallampalli RK, Li X, Jang JH, Kaminski T, Hoji A, Coon T, et al. Cigarette smoke exposure enhances transforming acidic coiled-coil-containing protein 2 turnover and thereby promotes emphysema. JCI Insight. 2020;5:e125895. 10.1172/jci.insight.125895 PMC7098723 31996486

[27] Ni J, Shen L, Xu L, Jin Q, Wang G (2021). 7-Ethoxyrosmanol alleviates hyperglycemia-induced vascular endothelial dysfunction by regulating FBXL7 expression. J Bioenerg Biomembr.

[28] Park HW, Dahlin A, Tse S, Duan QL, Schuemann B, Martinez FD (2014). Genetic predictors associated with improvement of asthma symptoms in response to inhaled corticosteroids. J Allergy Clin Immunol.

[29] Dahlin A, Sordillo JE, McGeachie M, Kelly RS, Tantisira KG, Lutz SM (2020). Genome-wide interaction study reveals age-dependent determinants of responsiveness to inhaled corticosteroids in individuals with asthma. PLoS ONE.

[30] Wu P, Wang K, Yang Q, Zhou J, Chen D, Ma J (2018). Identifying SNPs and candidate genes for three litter traits using single-step GWAS across six parities in Landrace and Large White pigs. Physiol Genomics.

[31] Bosch JA, Sumabat TM, Hafezi Y, Pellock BJ, Gandhi KD, Hariharan IK (2014). The Drosophila F-box protein Fbxl7 binds to the protocadherin fat and regulates Dachs localization and Hippo signaling. Elife..

[32] Rodrigues-Campos M, Thompson BJ (2014). The ubiquitin ligase FbxL7 regulates the Dachsous-Fat-Dachs system in Drosophila. Development..

[33] Kamran M, Long ZJ, Xu D, Lv SS, Liu B, Wang CL (2017). Aurora kinase A regulates Survivin stability through targeting FBXL7 in gastric cancer drug resistance and prognosis. Oncogenesis..

[34] Li M, Gao F, Yu X, Zhao Q, Zhou L, Liu W (2020). Promotion of ubiquitination-dependent survivin destruction contributes to xanthohumol-mediated tumor suppression and overcomes radioresistance in human oral squamous cell carcinoma. J Exp Clin Cancer Res.

[35] Chiu HW, Chang JS, Lin HY, Lee HH, Kuei CH, Lin CH, et al. FBXL7 upregulation predicts a poor prognosis and associates with a possible mechanism for paclitaxel resistance in ovarian cancer. J Clin Med. 2018;7:330. 10.3390/jcm7100330 PMC6209951 30301218

[36] Keskin O, Farzan N, Birben E, Akel H, Karaaslan C, Maitland-van der Zee AH (2019). Maitland-van der Zee AH, et al. Genetic associations of the response to inhaled corticosteroids in asthma: a systematic review. Clin Transl Allergy.

[37] Gautam Y, Afanador Y, Abebe T, Lopez JE, Mersha TB (2019). Genome-wide analysis revealed sex-specific gene expression in asthmatics. Hum Mol Genet.

[38] Forno E, Wang T, Qi C, Yan Q, Xu CJ, Boutaoui N (2019). DNA methylation in nasal epithelium, atopy, and atopic asthma in children: a genome-wide study. Lancet Respir Med.

[39] Tosto G, Fu H, Vardarajan BN, Lee JH, Cheng R, Reyes-Dumeyer D (2015). F-box/LRR-repeat protein 7 is genetically associated with Alzheimer’s disease. Ann Clin Transl Neurol.

[40] Cornejo-Garcia JA, Liou LB, Blanca-Lopez N, Dona I, Chen CH, Chou YC (2013). Genome-wide association study in NSAID-induced acute urticaria/angioedema in Spanish and Han Chinese populations. Pharmacogenomics..

[41] Long NP, Park S, Anh NH, Min JE, Yoon SJ, Kim HM, et al. Efficacy of integrating a novel 16-gene biomarker panel and intelligence classifiers for differential diagnosis of rheumatoid arthritis and osteoarthritis. J Clin Med. 2019;8:50. 10.3390/jcm8010050 PMC6352223 30621359

[42] Zhang X, Feng S, Fan Y, Luo Y, Jin L, Li S (2020). Identifying a comprehensive ceRNA network to reveal novel targets for the pathogenesis of Parkinson’s disease. Front Neurol.

[43] Liu S, Chen H, Ma W, Zhong Y, Liang Y, Gu L (2022). Non-coding RNAs and related molecules associated with form-deprivation myopia in mice. J Cell Mol Med.

[44] Wheatley SP, Altieri DC. Survivin at a glance. J Cell Sci. 2019;132:jcs223826. 10.1242/jcs.223826 PMC6467487 30948431

[45] Garg H, Suri P, Gupta JC, Talwar GP, Dubey S (2016). Survivin: a unique target for tumor therapy. Cancer Cell Int.

[46] Summy JM, Gallick GE (2003). Src family kinases in tumor progression and metastasis. Cancer Metastasis Rev.

[47] Ortiz MA, Mikhailova T, Li X, Porter BA, Bah A, Kotula L (2021). Src family kinases, adaptor proteins and the actin cytoskeleton in epithelial-to-mesenchymal transition. Cell Commun Signal.

[48] Moro L, Pagano M (2020). Epigenetic suppression of FBXL7 promotes metastasis. Mol Cell Oncol.

[49] Nieto MA (2002). The snail superfamily of zinc-finger transcription factors. Nat Rev Mol Cell Biol.

[50] Dong B, Wu Y. Epigenetic regulation and post-translational modifications of SNAI1 in cancer metastasis. Int J Mol Sci. 2021;22:11062. 10.3390/ijms222011062 PMC8538584 34681726

[51] Zhou BP, Deng J, Xia W, Xu J, Li YM, Gunduz M (2004). Dual regulation of Snail by GSK-3beta-mediated phosphorylation in control of epithelial-mesenchymal transition. Nat Cell Biol.

[52] Yook JI, Li XY, Ota I, Fearon ER, Weiss SJ (2005). Wnt-dependent regulation of the E-cadherin repressor snail. J Biol Chem.

[53] Sun R, Xie HY, Qian JX, Huang YN, Yang F, Zhang FL (2018). FBXO22 possesses both protumorigenic and antimetastatic roles in breast cancer progression. Cancer Res.

[54] Vinas-Castells R, Beltran M, Valls G, Gomez I, Garcia JM, Montserrat-Sentis B (2010). The hypoxia-controlled FBXL14 ubiquitin ligase targets SNAIL1 for proteasome degradation. J Biol Chem.

[55] Zheng H, Shen M, Zha YL, Li W, Wei Y, Blanco MA (2014). PKD1 phosphorylation-dependent degradation of SNAIL by SCF-FBXO11 regulates epithelial-mesenchymal transition and metastasis. Cancer Cell.

[56] Jin Y, Shenoy AK, Doernberg S, Chen H, Luo H, Shen H (2015). FBXO11 promotes ubiquitination of the Snail family of transcription factors in cancer progression and epidermal development. Cancer Lett.

[57] Vinas-Castells R, Frias A, Robles-Lanuza E, Zhang K, Longmore GD, Garcia de Herreros A (2014). Nuclear ubiquitination by FBXL5 modulates Snail1 DNA binding and stability. Nucleic Acids Res.

[58] Wu W, Ding H, Cao J, Zhang W (2015). FBXL5 inhibits metastasis of gastric cancer through suppressing Snail1. Cell Physiol Biochem.

[59] Zou S, Ma C, Yang F, Xu X, Jia J, Liu Z (2018). FBXO31 suppresses gastric cancer EMT by targeting snail1 for proteasomal degradation. Mol Cancer Res.

[60] Xu M, Zhu C, Zhao X, Chen C, Zhang H, Yuan H (2015). Atypical ubiquitin E3 ligase complex Skp1-Pam-Fbxo45 controls the core epithelial-to-mesenchymal transition-inducing transcription factors. Oncotarget..

[61] Zhang Y, Zhang X, Ye M, Jing P, Xiong J, Han Z (2018). FBW7 loss promotes epithelial-to-mesenchymal transition in non-small cell lung cancer through the stabilization of Snail protein. Cancer Lett.

[62] Xiao G, Li Y, Wang M, Li X, Qin S, Sun X (2018). FBXW7 suppresses epithelial-mesenchymal transition and chemo-resistance of non-small-cell lung cancer cells by targeting snai1 for ubiquitin-dependent degradation. Cell Prolif.

[63] Ha GH, Kim JL, Breuer EK (2013). Transforming acidic coiled-coil proteins (TACCs) in human cancer. Cancer Lett.

[64] Cheng S, Douglas-Jones A, Yang X, Mansel RE, Jiang WG (2010). Transforming acidic coiled-coil-containing protein 2 (TACC2) in human breast cancer, expression pattern and clinical/prognostic relevance. Cancer Genomics Proteom.

[65] Shakya M, Zhou A, Dai D, Zhong Q, Zhou Z, Zhang Y (2018). High expression of TACC2 in hepatocellular carcinoma is associated with poor prognosis. Cancer Biomark.

[66] Takayama K, Horie-Inoue K, Suzuki T, Urano T, Ikeda K, Fujimura T (2012). TACC2 is an androgen-responsive cell cycle regulator promoting androgen-mediated and castration-resistant growth of prostate cancer. Mol Endocrinol.

[67] Hanahan D (2022). Hallmarks of cancer: new dimensions. Cancer Discov.

[68] Kalpana G, Figy C, Yeung M, Yeung KC (2019). Reduced RhoA expression enhances breast cancer metastasis with a concomitant increase in CCR5 and CXCR4 chemokines signaling. Sci Rep.

[69] Jeong D, Park S, Kim H, Kim CJ, Ahn TS, Bae SB (2016). RhoA is associated with invasion and poor prognosis in colorectal cancer. Int J Oncol.

[70] Nam S, Kim JH, Lee DH (2019). RHOA in gastric cancer: functional roles and therapeutic potential. Front Genet.

[71] Moller LLV, Klip A, Sylow L. Rho GTPases-emerging regulators of glucose homeostasis and metabolic health. Cells. 2019;8:434. 10.3390/cells8050434 PMC6562660 31075957

[72] Tan MK, Lim HJ, Bennett EJ, Shi Y, Harper JW (2013). Parallel SCF adaptor capture proteomics reveals a role for SCFFBXL17 in NRF2 activation via BACH1 repressor turnover. Mol Cell.

[73] Takamitsu E, Otsuka M, Haebara T, Yano M, Matsuzaki K, Kobuchi H (2015). Identification of human N-myristoylated proteins from human complementary DNA resources by cell-free and cellular metabolic labeling analyses. PLoS ONE.

[74] Yuan M, Song ZH, Ying MD, Zhu H, He QJ, Yang B (2020). N-myristoylation: from cell biology to translational medicine. Acta Pharm Sin.

[75] Wang B, Dai T, Sun W, Wei Y, Ren J, Zhang L (2021). Protein N-myristoylation: functions and mechanisms in control of innate immunity. Cell Mol Immunol.

[76] Wang X, Pankratz VS, Fredericksen Z, Tarrell R, Karaus M, McGuffog L (2010). Common variants associated with breast cancer in genome-wide association studies are modifiers of breast cancer risk in BRCA1 and BRCA2 mutation carriers. Hum Mol Genet.

[77] Liu C, Zhang YH, Huang T, Cai Y (2017). Identification of transcription factors that may reprogram lung adenocarcinoma. Artif Intell Med.

[78] Tang Z, Li C, Kang B, Gao G, Li C, Zhang Z (2017). GEPIA: a web server for cancer and normal gene expression profiling and interactive analyses. Nucleic Acids Res.

